# Implementing nationwide measles supplemental immunization activities in Ethiopia in the context of COVID-19: process and lessons learnt

**DOI:** 10.11604/pamj.supp.2020.37.1.26614

**Published:** 2020-11-16

**Authors:** Mulat Nigus, Meseret Zelalem, Kibrom Abraham, Amsalu Shiferaw, Mekonnen Admassu, Balcha Masresha

**Affiliations:** 1 Federal Ministry of Health, Addis Ababa, Ethiopia,; 2 United Nations Children's Fund (UNICEF), Addis Ababa, Ethiopia,; 3 World Health Organization, Addis Ababa, Ethiopia; 4 World Health Organization, Regional Office for Africa, Brazzaville, Congo

**Keywords:** Measles, immunization, COVID-19, vaccination, Ethiopia, Africa, supplemental immunization activity

## Abstract

The COVID-19 pandemic has disrupted immunization activities in many countries, causing declines in the delivery of routine doses of antigens, and the postponement of scheduled supplemental immunization activities (SIAs). Following the declaration of the pandemic, Ethiopia postponed nationwide follow-up measles preventive vaccination campaign which was scheduled for April 2020. The disruptions to routine services and the postponement of the SIAs increased the risk for measles outbreaks. The national authorities, in consultation with the secretariat of the National COVID-19 Pandemic Prevention and Control Ministerial Coordination Committee, subnational level authorities, technical partner agencies and stakeholders, reviewed the risks for measles outbreaks and decided to implement the nationwide measles SIAs, with strict implementation of COVID prevention measures. The revised micro-plans accommodated the additional human resource and logistics needs for COVID prevention, for which partner resources were mobilized to fill the gaps. The key SIAs preparatory and implementation activities including training, logistics, social mobilization, service delivery and supervision were modified to take into consideration the COVID context. Infection prevention and control supplies were procured and distributed as a package with the bundled vaccines and other supplies. The SIAs were completed in July 2020 and reached 102.8% administrative coverage nationwide, with 78% of the 1123 woredas attaining the target of 95% coverage. The strong commitment of the leadership, the coordination role of the national and regional COVID prevention and control taskforces, the engagement of community leaders, the use of multi-channel communication, the timely availability of additional resources and modification of the service delivery approaches contributed to the success of the SIAs.

## Project evaluation

Ethiopia has been implementing the accelerated measles control strategies since 2004 and adopted the African Regional measles elimination goal in 2011, aiming for the achievement of the set targets by 2020 [1]. The country implemented efforts to improve routine vaccination coverage with the first dose measles vaccine (MCV1) and has been conducting intensified laboratory-supported surveillance for measles. To reach unvaccinated children and close the immunity gaps among young children, Ethiopia has been conducting periodic preventive mass vaccination campaigns (also referred to as “Supplemental Immunization Activities”) [2-4]. In addition, Ethiopia introduced the second dose measles vaccine (MCV2) in the routine immunization program in February 2019.

The World Health Organization (WHO) declared the COVID-19 pandemic on 11^th^ March 2020 [5]. By 30^th^ June 2020, all 47 countries in the African Region of the WHO were affected with a cumulative total of 303,986 COVID-19 cases and 6,155 deaths; Ethiopia had 5,846 confirmed COVID cases and 103 deaths [6]. Public fear of catching COVID-19 caused a decline of demand, while the mobilization of response measures including the decisions to limit movement that was imposed in many countries resulted in declines in the utilization of regular preventive and curative health services. Many countries reported variable degrees of disruption of routine immunization services and there was also a postponement of previously scheduled mass vaccination campaigns against measles, yellow fever and polio in a few countries [7-9]. Countries in the African region with weak immunization systems had a significant decline in the number of vaccination doses provided in the first three months after the declaration of the COVID-19 pandemic [10]. Models show the impact on maternal and child health, with increased mortality as a result of the disruption to multiple essential interventions [11]. One of the programmatic areas where reductions in vaccination coverage can cause significant mortality is measles immunization [12]. There is a significant risk of losing the disease control gains made in the last two decades if these disruptions continue for a long period of time. The Ebola outbreak in West Africa in 2014-2015 significantly affected measles vaccination coverage rates, particularly in Guinea and Liberia, where more than 25% decline in routine immunization coverage was documented in both years, as compared to the pre-Ebola years. This led to high measles incidence that persisted for at least two years after the end of the Ebola outbreak in both countries [13]. An outbreak of circulating vaccine derived polio virus (cVDPV) type-2 was also documented in Guinea following the Ebola outbreak [14].

With these lessons in mind, WHO developed technical guidelines in March 2020 to assist countries with maintaining essential health interventions including immunization, by integrating specific measures to protect the health workers and clients, assure the safety of service and prevent COVID transmission in health care settings [15,16]. In May 2020, WHO issued a decision making framework to guide countries as they consider mounting mass vaccination activities [17].

**Justification for the periodic measles follow-up supplemental immunization activities:** according to the WHO-UNICEF coverage estimates, Ethiopia´s MCV1 coverage ranged between 68% in 2011 (the year of the adoption of the Regional measles elimination goal) and 58% in 2019 [18]. According to the Ethiopia Demographic Health (DHS) Survey in 2016, MCV1 coverage was 59% at national level, with provincial level MCV1 coverage ranging from 30.1% in Afar Region to 93.1% in Addis Ababa [19]. The country conducted the last measles Supplemental Immunization Activities (SIAs) in 2017, and attained administrative coverage of 96.5%, while the national coverage was 93.2% (95% Confidence Interval of 92.1%-94.0%) in the post-campaign survey [20,21]. With these coverage levels, it is estimated that there will be an accumulation of susceptible young children reaching the size of one birth cohort every 2-3 years, leading to the possibility of large outbreaks [22,23]. Data from measles surveillance indicated that Ethiopia reported 1597 confirmed measles cases in 2018, and 3998 cases in 2019. In the months January to June of 2020, there were a total of 1846 confirmed measles cases reported from across all Regions, with an annualized incidence of 34 cases per million population. Seventy eight percent of these confirmed cases from the first half of 2020 were unvaccinated. Nearly half (47%) were in the age group less than 5 years of age and 22% were in the age group 5-9 years [24].

Ethiopia had planned to conduct nationwide measles SIAs in 2020, targeting children aged 9-59 months, for which partner funding was already secured in 2019. The country started preparations for the SIAs since July 2019 with a view to implement the SIAs in April 2020. The required bundled vaccines were in country by March 2020. This manuscript attempts to document the process taken by the Federal Ministry of Health (FMoH) to implement the SIAs within the context of COVID-19, and the outcomes thereof. We reviewed the program data, the periodic programmatic updates on the preparations towards the campaign implementation, and the available official documentation on the nationwide measles SIAs.

**The decision making on the measles SIAs in the COVID context:** the first cases of COVID-19 were reported in Ethiopia on 13^th^ March 2020. By 8^th^ April 2020, there were a total of 52 confirmed cases and 1 death [25]. On 8^th^ April, the government imposed a State of Emergency which included a prohibition of public gatherings or meetings, and limited the amount of persons seated together in public spaces and public transportation [26]. This proclamation was followed by a postponement of all mass vaccination activities including the measles SIAs scheduled for April 2020. In May 2020, the Federal Ministry of Health undertook several consultations with the Secretariat of the National COVID-19 Pandemic Prevention and Control Ministerial Coordination Committee, the Regional Health Bureaus, the immunisation technical partners, the National Immunization Technical Advisory Group (NITAG), and other senior health authorities about the risks for measles outbreaks, the modelling of the impact of COVID-19 on child mortality, and the requirements to conduct measles SIAs safely.

The NITAG helped to evaluate the risks against the benefits of conducting the SIAs. The evaluation of risks looked at the following local data-the estimated accumulation of children susceptible to measles, the seasonality of measles transmission in the country, the incidence of measles from the surveillance data, the COVID transmission patterns and trends, information from modelling of measles incidence in the COVID context. On 15^th^ May 2020, the NITAG recommended to conduct nationwide measles SIAs in order to avoid outbreaks, considering the already large number of unprotected children, which will further increase with the disruptions caused by the COVID-19 pandemic and the control measures in place. Regarding the timing of the SIAs, the NITAG recommended that the SIAs should be implemented as soon as possible before the country reached a phase of high-intensity and widespread COVID-19 transmission which would make it impossible to implement any mass vaccination interventions. In addition, the advisory group emphasized the need to ensure that COVID-19 infection prevention and control measures are put in place as a prerequisite before conducting SIAs.

The Ministry of Health worked with the secretariat of the National COVID-19 Pandemic Prevention and Control Ministerial Coordination Committee, and secured operational approval to implement the SIAs with adherence to the State of Emergency regulations and implementing infection prevention and control (IPC) measures. The National Measles SIAs Coordination Command Post was established, chaired by the State Minister of Health, and conducted regular coordination meetings until the start of the SIAs, which were held daily during the SIAs implementation period.

**Operational preparations for the nationwide measles SIAs:** as per WHO recommendations, the following key elements were put in place while considering to conduct mass vaccination in the context of COVID-19 [15]. i) Evaluation of the country's capacity to implement a mass vaccination campaign safely and effectively under the strain of COVID-19 (including the adequacy of human resources, logistical and transport barriers, flow of material and financial needs); ii) listing of actions to be taken to conduct high-quality and safe vaccination campaigns without undue harm to health workers and the community; iii) understanding the demand of the community in the midst COVID-19 and the need to engage community leaders in the planning and implementation; iv) coordinating with COVID-19 task force to reflect on the strategies that need to be applied to infection prevention and control measures; v) ensuring the availability of adequate infection prevention and control supplies, including personal protective equipment (PPE) for mitigation of COVID-19 transmission; vi) training of health workers and volunteers to strictly adhere to infection, prevention and control recommendations while organizing vaccination sites and during vaccination; vii) confirming strong and effective supervision and monitoring mechanism is in place at all levels. The preparatory activities took these points into consideration, and the necessary modifications were accommodated into micro-planning, the training of health workers, communication and social mobilization, as well as logistics preparations. The national immunization program and partners worked to amend the SIAs plan and budget to include the costs for the additional human resources, additional days of service delivery, as well as the IPC supplies required.

**Revision of micro-plans:** as part of the initial preparation for the SIAs, district microplanning had been completed before the onset of COVID-19. However, considering the changes needed at the operational level, micro-planning templates were revised to include the human resources and supplies required for COVID-19 infection prevention and control. The composition of the vaccination team was revised to include a total of six persons (three health workers and three volunteers). One health worker was added to the team to handle COVID-19 screening and awareness raising, while one volunteer was added to monitor physical distancing and hand washing or sanitization at the vaccination site. The revision of the micro-plan template also included an extension of the duration of SIAs implementation from the initial 7 days to 10 days, as well as a proposed reduction of the expected number of children to be reached by each team from 350 to 245 per day in densely populated areas, and a reduction from 250 to 175 children per team per day in sparsely populated areas. Woredas (the equivalent of districts) were supported to revise their micro-plans accordingly, following which the plans were validated at national level. The finalized woreda level micro-plans increased the total number of vaccination teams nationwide from 6,106 in the initial set of microplans to 6,332 in the final version, and the number of required health workers from the initial number of 12,212 to a total of 18,996.

**Resource mobilization:** additional financial resources totaling USD 7,427,000 were mobilized from partners to cover expenses required for the safe implementation of the mass vaccination activity. The GAVI Alliance approved reprogramming of funds from the Health Systems Strengthening envelope for COVID-19 prevention and control. In addition, the US Centers for Disease Control (CDC) contributed funds to cover the needs for the additional human resources, as well as training and procurement of IPC supplies, while the Irish government contributed funds for the procurement of facemasks. All immunization partners provided technical support throughout the preparation and implementation of the SIAs. Besides, partners working at sub-national level and government sector offices provided additional human resource and means of transportation during the SIAs.

**Training of health workers and volunteers:** the measles SIA implementation field guide was revised to include the necessary changes as well as elements of risk communication in the context of COVID-19. The guideline was used for a series of virtual training sessions for trainers and supervisors from the national and sub-national levels. The cascaded training of health workers and vaccination teams at woreda and health facility levels was conducted face-to-face, in small size sessions of 10-20 participants, taking the necessary precautions including face masks, hand sanitising and physical distancing. The training in these face-to-face sessions were practical in nature, and emphasized the various measures put in place to prevent COVID-19 transmission at the service delivery site in addition to the standard topics including the roles of team members, safe handling of vaccines and vaccination, recognition and management of adverse events, use of monitoring tools. Volunteers were similarly trained at woreda level, with an emphasis on the organization of the service delivery site, the roles of volunteers within the teams, and actions to take to prevent COVID-19 transmission.

**Advocacy, communication and social mobilization:** the political and technical leadership at national level provided guidance for the implementation of measles SIAs according to the COVID-19 prevention measures specified in the State of emergency declaration. In addition, the Regional COVID-19 Pandemic Response Emergency Operation Centers coordinated the measles SIAs at the regional level. This facilitated the coordination, decision making and overall political and operational support by the administrative authorities at Zonal and Woreda levels. Social mobilization was done through briefings from local health authorities and health workers, as well as messages from religious and community leaders, and spot announcements which were broadcast via major television, FM radio, as well as community radio channels. These were aired starting 5 days before the start of the SIAs and throughout the duration of the SIAs. Media briefings were provided on the need to maintain essential services by the leadership from the FMOH. The concerned FMOH Director and the immunization program manager appeared on national radio and television channels and provided messages on the SIAs. All of the Regions used local media extensively to provide similar messages. Broadcasted massages included announcements on the nature, the dates and target age for the campaign, as well as precautionary measures to be taken by clients at the vaccination site. In addition, the national level developed posters and brochures in four local languages and distributed them to the Regions. Once the campaign started, town criers mobilized communities moving across towns and villages using megaphones. Community and religious leaders, as well as members of the health development team and volunteer youth supported grass roots social mobilization, transporting of bundled vaccines, maintaining physical distancing at service delivery posts, and preparing hand-washing setup and encouraging clients to wash hands. Very good turnout was reported in many places starting from the first day of the SIAs. There was no major challenge reported with regards to hesitancy against the SIAs.

**Logistics:** the shipment of bundled measles vaccine into the country was already done in March 2020 and was not affected by the COVID-19 pandemic. However, infection prevention and control supplies and adrenaline ampoules (as part of the kit for the management of adverse events following immunization) were procured and delivered through an expedited order. Based on the micro-plans, the Ethiopian Pharmaceutical Supply Agency (EPSA) and all 17 EPSA hubs prepared detailed logistics plans and managed to distribute 16,316,942 doses of vaccines and other supplies to all woredas. In most cases, bundled vaccines, monitoring tools, facemasks and hand sanitizers, as well as printed mobilization materials (posters and brochures) were distributed as a package based on the micro-plans. The distribution took place in the last half of June 2020, and started from remote woredas to facilitate logistics and avoid possible disruptions with the rainy season. During this nationwide measles SIAs, among other inputs, the national government procured and distributed Adrenaline to the woreda and health facility level. A few woredas reported delayed supply of adrenaline and facemasks in the initial days of the SIAs. The Woredas organized to borrow these supplies from existing health facility stocks where possible or from private clinics. Various government sector offices and non-governmental organisations provided additional vehicles to assist in the last mile distribution of vaccines, the movement of vaccinators and supervisors. While the recommended microplanning standards were clear with regards to the team composition and daily targets per team, the actual microplans and field operations considered the density of local settlements, geographic access, the local pattern of COVID-19 transmission, the security situation in the area and other local factors.

**Readiness assessment:** the SIAs readiness assessment tool was adapted from the WHO field guide for SIA planning and implementation [27]. The assessment includes the preparedness status with regards to planning and coordination, vaccine cold chain, logistics, communication and social mobilization, monitoring and evaluation, with indicators for the national and woreda levels. The adaptation of the tool also included elements related to the logistics of COVID prevention. The readiness for the measles SIAs was assessed at national level, and in 204 woredas (20% of all woredas) across 9 Regions. Woredas were selected in each Region based on their routine immunization performance, surveillance performance, previous SIAs performance and the presence of refugee or displaced populations. Assessments were conducted at four different points in time by the Regional health bureau teams and local partners. The results of readiness assessment were used to drive the preparations, and also to decide on the start of the SIAs in each Region.

**COVID-19 prevention measures applied during measles SIAs:** the organization of vaccination sites was done in such a way as to allow measures for COVID-19 infection prevention and control. Fixed and temporary static vaccination sites were set up in well-ventilated open areas to enable proper physical distancing as much as possible, while queuing for the services. The number of family members accompanying a child to the vaccination post was limited to one. The flow of clients was organized to avoid crowding. In addition, a mobile fixed approach was utilized in many areas where, after the first few days of service at a fixed site, vaccination teams inform the community and move between clusters of villages to bring the services closer to the community. Awareness raising on COVID-19 prevention methods was conducted regularly in each vaccination site.

A total of 394,831 facemasks and 341,102 bottles of hand sanitizers (sizes of 100ml each) were procured for the SIAs. The provision of masks and sanitisers was done in such a way to provide 1 mask for each vaccination team member per day for the 10 days; and five bottles of sanitizers were issued per team for the 10 days campaign duration. Face masks and sanitizers were distributed along with the bundled vaccines from the central stores and logistics hubs to the district and health facility level. In addition to sanitizers distributed through EPSA, some universities produced sanitisers and provided them to health facilities. Hand washing stations were available at some vaccination sites. In woredas where the supply of facemasks was delayed or inadequate, health authorities prioritized the SIAs and mobilized facemasks from available stocks. All health workers and volunteers were required to wear facemasks, while health workers conducting vaccination were instructed to sanitise their hands between clients. Caretakers in urban areas bringing children for vaccination were mostly wearing surgical facemasks, while in rural areas caretakers used standard surgical facemasks or improvised cloth masks. However, it was noted that in hot weather areas, clients were not strictly adhering to the proper use of facemasks. Volunteers encouraged clients to sanitise their hands or to wash hands at vaccination sites where hand washing facilities were available. In each vaccination site, a health worker was assigned to do screening of clients, by checking temperature using infrared thermometers. In vaccination sites where there was no infrared thermometer, health workers screened by asking for symptoms such as fever, cough, and/or shortness of breath.

**Monitoring and supervision of the SIAs:** prior to the start of the campaign, trained supervisors and technical assistants from the national and provincial level, as well as partner agencies were deployed to support the SIAs implementation. Supervisors from various levels visited the service delivery sites during the campaign. With the exception of a few vaccination sites, where disruption of supplies was addressed locally, no major shortage of vaccines was reported. Supervisors checked cold chain equipment, the vaccine vial monitors (VVM), as well as the vaccine handling practices, recording of doses in the monitoring tools provided, and measures in place to minimize the risks for transmission of COVID-19. In most woredas, supervisors were tasked to conduct rapid convenience monitoring (RCM) of the SIAs in the last days of the exercise, specifically targeting geographic areas with high likelihood of coverage gaps. If there were more than 5% of children missed in any area, logistical and operational arrangements were done locally to identify and vaccinate missed children before the end of the SIAs. In some areas, this data was used to organize mop-up vaccination.

**The outcome of the SIAs:** five Regions (Amhara, Afar, Benshangul Gumuz, Tigray and Gambella) launched the campaign as per schedule on 30^th^ June 2020. The rest of the Regions delayed their starting date from 3-7 days, as a result of the security instability at the time, but also the delays in delivering the inputs (mostly supplies of adrenaline ampoules, facemasks and other dry goods). A few woredas that started late completed the mass vaccination campaign by 21^th^ July 2020. As dates of campaigns were extended, mop-up activities were completed in areas where coverage gaps were noted from the administrative coverage and monitoring visits. The administrative coverage at national level was 102.8%. Coverage ranged from 95.2% of the target in Harari region to 126% in Addis Ababa (Table 1). Out of all the vaccinated children, it is noted that 1,367,014 (9.4%) children were aged 9-11 months while 13,210,245 (90.6%) were between 12 and 59 months of age. Out of the 1123 woredas, special woredas and special towns in the country, 882 (78.5%) have attained at least 95% administrative coverage in the SIAs. The SIAs reached 645,294 children (4.4% of the total vaccinated) who had not received any dose of measles vaccine before (also referred to as “zero-dose children”), as well as 92,392 eligible children among the refugee community in Somali, Benishangul Gumuz and Gambella Regions. This proportion of zero-dose children is consistent with the findings from the Rapid Convenience Monitoring. However, considering the levels of routine immunization coverage in the country, it is expected that many Regions would reach a larger proportion of zero dose children than reported in the SIAs, raising the need to ensure more strict monitoring of the proportion of zero-dose children in future SIAs.

**Table 1:** administrative coverage of the nationwide measles SIAs, Ethiopia, July 2020

Regions	Target population	Number of children vaccinated	Coverage	Number of Zero dose children reached	% of zero dose children reached
Addis Ababa	335,212	423,613	**126.4%**	5,275	**1.2%**
Afar	266,208	262,293	**98.5%**	23,529	**9.0%**
Amhara	2,697,440	2,714,439	**100.6%**	64,966	**2.4%**
Benishangul Gumuz	155,565	154,925	**99.6%**	2,655	**1.7%**
Dire Dawa	63,666	63,922	**100.4%**	1,877	**2.9%**
Gambella	131,047	125,337	**95.6%**	1,227	**1.0%**
Harari	36,281	34,556	**95.2%**	213	**0.6%**
Oromia	5,817,924	6,133,343	**105.4%**	340,532	**5.6%**
Sidama	640,948	660,865	**103.1%**	3,946	**0.6%**
SNNPR	2,433,560	2,451,129	**100.7%**	75,812	**3.1%**
Somali	889,030	858,904	**96.6%**	116,554	**13.6%**
Tigray	714,262	693,933	**97.2%**	8,708	**1.3%**
**National**	**14,181,143**	**14,577,259**	**102.8%**	**645,294**	**4.4%**

Six Regions (Addis Ababa, Amhara, Benishangul Gumuz, Gambella, Sidama, Tigray) compiled and shared the results of the RCM to the national level. The monitoring exercise was conducted in 1,298 Kebeles (the lowest administrative unit) in 243 woredas across these 6 Regions. The vaccination status of 27,353 eligible children was checked and only 880 children (3.2%) were found not to have been vaccinated during the SIAs in these areas. The measles SIAs has helped to put the routine immunization system high on the agenda at all levels, and to revitalize task forces. The SIAs mobilized a considerable number of health workers and community volunteers who were not actively engaged due to COVID-19 related state of emergency. The bottom-up micro-planning helped to map all villages and demarcate catchment areas, for later use in the routine immunization system. The SIAs training was an opportunity to improve knowledge, skill and practice of health workers. in addition, most woredas conducted cold chain inventory in their catchment areas and maintained refrigerators which will continue to be used in the routine immunization program. The SIAs reached a considerable number of children who were not vaccinated before. The SIAs was used to mobilize the target children for MCV2 vaccination and other missed vaccination doses.

**Adverse events following immunization (AEFI):** during the SIAs, 22 cases of adverse events following immunization were reported, of which 4 were severe cases. There were 2 reported deaths in Gambella Region and 2 deaths in Amhara region among these reported AEFI cases. The teams investigating these deaths have taken the necessary clinical and programmatic information at the time, and the National AEFI Committee has also launched its own investigation. The causality assessment results have not been officially released as of mid-October 2020. The country had set up a COVID surveillance system to identify imported and local transmission of the virus, including among health workers. There was no specific system in place to track the occurrence of COVID cases among vaccination teams or among persons who visited vaccination sites. However, the COVID surveillance system did not indicate any increase in COVID cases among the general population nor among health workers in the 3 weeks following the conclusion of the SIAs.

On 7^th^ August 2020, Ethiopia launched a one-month period of intensive COVID testing campaign, with a view to better understand the extent of local transmission, and use the opportunity for awareness raising [28]. The COVID testing campaign increased the number of daily COVID tests in the country more than 3-fold to an average of 21,000 tests per day as compared to the situation at the end of July 2020. The widespread testing brought the 7-day rolling average of COVID-positive cases from 612 per day across the country on 6^th^ August to 1566 COVID positive cases by 27^th^ August 2020. These numbers came down to a 7-day rolling average of 593 COVID positive cases per day by 18^th^ September 2020 [29].

**Challenges:** some of the challenges faced during this nationwide campaign include the following; i) Competing priorities as a result of the timing of the SIAs overlapping with the end of the fiscal year, the COVID-19 pandemic, the start of the farming season in the central highlands; ii) A burst of political unrest mainly in Oromia and Addis Ababa that started on 30^th^ June 2020 was followed by challenges of cross-country travel around Addis Ababa and surrounding zones of Oromia Region for a few days; iii) The internet shutdown following the unrest occurred over the 10-day period of the SIAs, and interrupted the mass mobile messaging platforms (on Telegram group messaging App) that were set up for the coordination of the SIAs, as well as the real-time mobile supervisory data capture system (using One Data Kit software); iv) Delays in the disbursement of funds to the woreda level; v) Delays in the distribution of face masks and adrenaline ampoules to some Regions. Most of the challenges were overcome through regular communication and coordination between the different levels, as well as proactive leadership and coordination from the federal level. When SIAs operational funds were delayed, available funds were mobilized for operations and for the local purchase of AEFI medications to be later reimbursed. Facemasks from available stocks were mobilized when supplies were delayed. Areas experiencing political unrest started the campaign implementation after a few days delay and worked through 10 days in order to reach unreached children. In the absence of internet connection, the immunization program managers at all levels and the vaccination teams utilized short text messaging services (SMS) and telephone calls to coordinate and share administrative coverage data and other information on the conduct of the SIAs. Mobile fixed vaccination strategies helped to bring services closer to the community.

**Lessons learnt and way forward:** the decision making, coordination and implementation of the nationwide measles SIAs was facilitated as a result of multiple factors: i) The risk-benefit analysis based on available evidence led to an informed decision; ii) Wider consultation among stakeholders including the secretariat of the National COVID-19 Pandemic Prevention and Control Ministerial Coordination Committee, the NITAG and partner agencies helped to gain overall support; iii) Strong commitment of the political-administrative leadership as well as the public health leaders and health workers; iv) Assuring strong public demand through multi-channel communication, social mobilization and community engagement; v) Timely partner support to mobilise additional resources to cover the financial gaps helped address the COVID-19 prevention activities; vi) Assuring decongestion during the mass vaccination exercise by extending the duration of service delivery from 7 to 10 days, delivering services in open areas, implementing physical distancing and IPC guidelines, and adopting a mobile-fixed approach where vaccinators move their posts to get closer to settlements on different days; vii) The availability of digital communication means was very helpful to conduct technical consultations, trainings, coordination with partners and stakeholders, as well as coordination and communication with the teams in the field; viii) The availability of adequate PPE and IPC supplies. Following this successful SIAs, Ethiopia will validate the administrative coverage by doing a cluster survey. In the coming years, Ethiopia will have to continue monitoring routine immunization coverage and put efforts into improving MCV1 as well as MCV2 coverage, with a focus on areas with large number of unvaccinated children. The national immunization program will also work to implement efforts for the Periodic Intensification of Routine Immunization, and also identify innovative means to tailor services so as to reach unreached children. Expanding the age for the delivery of primary and booster doses beyond 24 months is critical so as to offer the opportunity for unvaccinated older children to be vaccinated whenever they come into contact with health services.

Improving immunization coverage calls for strengthening the implementation of the fundamental approaches of reaching every district, minimizing missed opportunities, integrating service delivery so that all family visits to health facilities are optimized, and using measles coverage and surveillance data in order to map out and address immunity gaps. In the course of the last 15 years, measles surveillance has uncovered the extent of rubella transmission in Ethiopia, indicating the need to conduct a retrospective review or to set up a prospective system of surveillance of congenital rubella syndrome (CRS) in the country. The available evidence on rubella transmission helps in the decision making to introduce rubella vaccine. The next measles preventive SIAs in Ethiopia is expected to use measles-rubella (MR) vaccine and will target a wide age range of children as per the WHO recommendations for the initial introduction of rubella vaccine.
